# Preoperative diagnosis and safe surgical approach in gallbladder amyloidosis: a case report

**DOI:** 10.1186/s40792-024-01897-8

**Published:** 2024-04-18

**Authors:** Makoto Shinohara, Masakazu Hashimoto, Yoshihito Kitamura, Keigo Nakashima, Michinori Hamaoka, Masashi Miguchi, Toshihiro Misumi, Nobuaki Fujikuni, Satoshi Ikeda, Yasuhiro Matsugu, Yui Hattori, Takashi Nishisaka, Hideki Nakahara

**Affiliations:** 1 Department of Gastroenterological Surgery, Hiroshima Prefectural Hospital, 1-5-54 Ujinakannda, Minami-ku, Hiroshima, 734-8530 Japan; 2 Department of Pathology and Laboratory Medicine, Hiroshima Prefectural Hospital, Hiroshima, Japan

**Keywords:** Gallbladder amyloidosis, Acute cholecystitis, Gallbladder dyskinesia

## Abstract

**Background:**

Preoperative diagnosis of gallbladder amyloidosis is usually difficult. In our case, the patient exhibited gallbladder dyskinesia, which led us to suspect cholecystic amyloidosis. We were able to safely perform surgery before cholecystitis onset.

**Case presentation:**

A 59-year-old male patient with a history of multiple myeloma and cardiac amyloidosis presented to our hospital with a chief complaint of epicardial pain. Abdominal ultrasonography and computed tomography revealed an enlarged gallbladder and biliary sludge without any specific imaging findings of cholecystitis. After percutaneous transhepatic gallbladder aspiration (PTGBA), the patient experienced recurrent bile retention and right upper quadrant pain. Flopropione was effective in relieving these symptoms. Based on his symptoms and laboratory findings, we diagnosed the patient with dyskinesia of the gallbladder. Considering his medical history, we suspected that it was caused by amyloidosis of the gallbladder. A laparoscopic cholecystectomy was performed. The histopathological examination showed amyloid deposits in the gallbladder mucosa, from the intrinsic layer to the submucosa, and in the peripheral nerves of the gallbladder neck. The patient was discharged on postoperative day 5 and has had no recurrence of abdominal pain since then.

**Conclusion:**

In our case, gallbladder dyskinesia symptoms led us to suspect gallbladder amyloidosis. We safely surgically treated the patient before cholecystitis onset.

## Background

Amyloidosis is a disorder caused by misfolded proteins that form insoluble fibrils deposited in the extracellular space of tissues, ultimately causing progressive organ dysfunction [1]. Although amyloidosis can be local; it is mostly a systemic disease that can involve almost every organ in the body, including the heart, kidneys, gastrointestinal tract, and nervous system [2, 3]. Gallbladder amyloidosis is a rare condition; its preoperative diagnosis is exceptionally challenging, as it typically manifests with symptoms resembling acute cholecystitis [4, 5]. In our case, the patient with cardiac amyloidosis exhibited gallbladder dyskinesia, which led us to suspect cholecystic amyloidosis. We were able to safely perform surgery before cholecystitis onset.

## Case presentation

A 59-year-old male patient presented to our hospital with epigastric pain. His medical history included multiple myeloma and cardiac amyloidosis; he was undergoing chemotherapy. On physical examinations, the patient had a body temperature of 37.1 ℃ and his abdomen was soft and flat, with right-upper-quadrant tenderness and a mild Murphy’s sign.

**Fig. 1:**
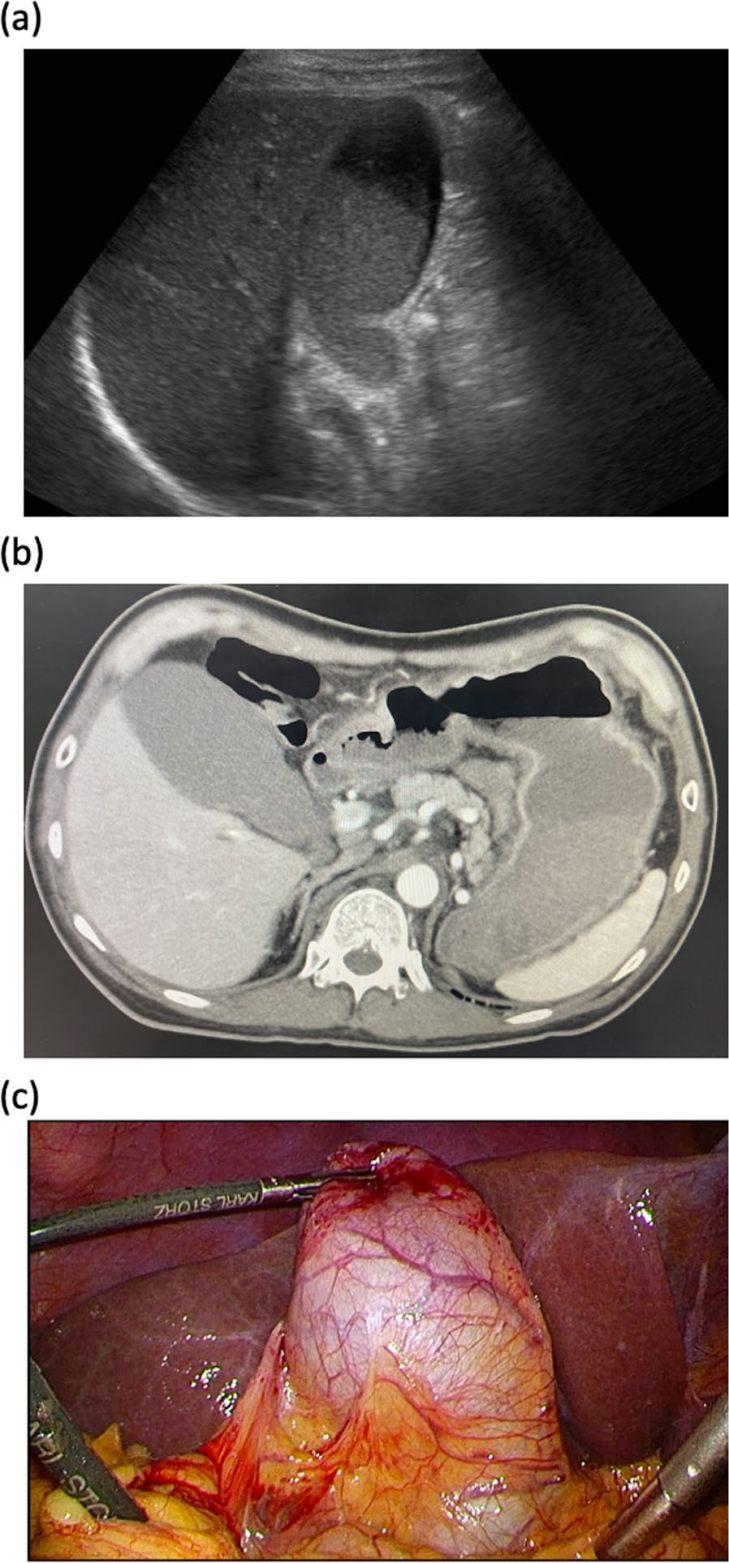
**a** Abdominal ultrasonography. No gallstones or wall thickening are observed. Bile sludge is detected in the gallbladder. Contrast-enhanced abdominal tomography. The gallbladder is mildly enlarged; however, no wall thickening or gallstone formation is observed. **b** Surgical findings. The gallbladder is mildly enlarged; however, no thickening is observed, and there is little evidence of neovascularization and tissue fragility

Laboratory test results indicated an elevated white blood cell count of 13,400/μL and a C-reactive protein level of 0.33 mg/dL. There were no apparent abnormalities observed in blood testing for the liver or biliary functions. Abdominal ultrasonography revealed an enlarged gallbladder with biliary sludge without evidence of wall thickening or calculi (Figs. 1a, b). Upper gastrointestinal endoscopy did not reveal any abnormal findings. Endoscopic retrograde cholangiopancreatography was performed with planned endoscopic naso-gallbladder drainage (ENGBD). Since ENGBD was difficult to place, endoscopic nasobiliary drainage (ENBD) and endoscopic sphincterotomy were performed for suspected biliary mud in the common bile duct. After ENBD, he underwent percutaneous transhepatic gallbladder aspiration (PTGBA) due to persistent bile mud accumulation and right upper quadrant pain.

**Fig. 2:**
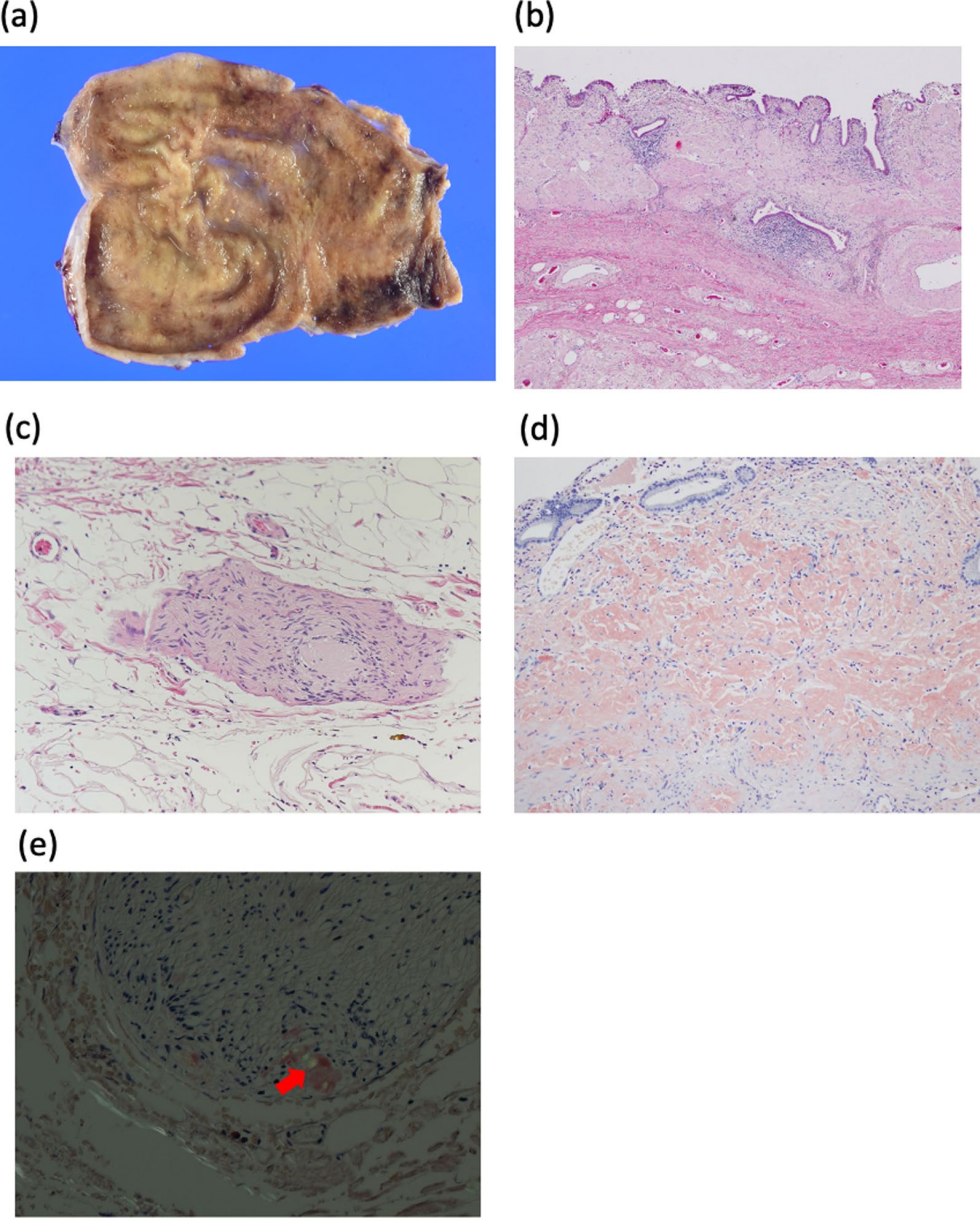
Macroscopic findings. **a** Gallstones are absent but there is bile sludge in the gallbladder. Microscopic findings. **b** Hematoxylin and eosin staining showing diffuse deposition of eosinophilic material from the mucosal lamina propria to the subserosal layer. **c** Hematoxylin and eosin staining showing amyloid deposition in the peripheral nerves of the gallbladder neck. **d** Salmon pink-colored eosinophilic deposition in the mucosal lamina propria and muscularis propria [direct fast scarlet (DFS) staining]). **e** DFS staining shows the deposition exhibits apple-green birefringence under polarized light (←) in the peripheral nerves

After PTGBA, the patient continued to experience a reservoir of bile sludge and recurrent right upper quadrant pain. However, oral flopropione administration at the time of pain was remarkably effective in reducing these symptoms. Based on the efficacy of flopropione in managing colic attacks and presence of gallbladder dysfunction with a history of amyloidosis, we suspected that the patient had gallbladder dyskinesia due to gallbladder amyloidosis. The patient responded to induction chemotherapy for multiple myeloma without adverse events. However, his cardiac function was impaired, with a left ventricular ejection fraction of 36%, an overall decrease in wall motion, and an elevated NT-proBNP up to 3652 pg/mL. Surgery was suggested. However, the patient preferred to be followed up because his symptoms were stable, being concerned about worsening heart failure due to surgical invasion. The symptoms settled down after that. One year later, he experienced right upper abdominal pain and came to see the physician. His general condition was stable. Therefore, we decided to perform laparoscopic cholecystectomy for therapeutic and diagnostic purposes. During the surgical procedure, the gallbladder was mildly enlarged. However, no thickening was observed, and there was little evidence of neovascularization or tissue fragility (Fig. 1c). The surgery was completed without any complications, with blood loss of 28 mL and surgical time of 106 min. Examination of the resected specimen revealed an absence of calculi in the gallbladder. Macroscopic analysis of the resected gallbladder demonstrated longitudinal sectioning, revealing diffuse deposition of eosinophilic material from the mucosal lamina propria to the subserosal layer, as evidenced by hematoxylin and eosin staining. Direct fast scarlet staining revealed a salmon pinkish coloration, indicating amyloid deposition in the mucosal lamina propria, muscularis propria, and peripheral nerves. Amyloid deposition was observed in the peripheral nerves of the subserosal layer of the gallbladder neck (Fig. 2). The patient's postoperative course was uneventful, leading to discharge on postoperative day 5. To date, no recurrence of the abdominal pain has been observed. In this case, preoperative suspicion of gallbladder amyloidosis allowed for surgically treating the patient while being in good general condition before developing cholecystitis.

## Discussion

In this case, gallbladder amyloidosis was suspected preoperatively based on the symptoms of gallbladder dyskinesia in a patient with a background of amyloidosis. We performed surgery electively and safely before cholecystitis onset in a patient with preexisting amyloidosis.

Preoperative diagnosis of gallbladder amyloidosis is extremely difficult [6]. In case reports to date, the diagnosis has been confirmed using postoperative histopathological specimens. The reason is that gallbladder amyloidosis is difficult to differentiate from commonly experienced cholecystitis because there are no characteristic imaging findings. In addition, the disease is rare [6, 7]. A search in PubMed using the MeSH keyterms “amyloidosis” and “gallbladder” revealed six case reports in English literature within the period 2000–2022. These cases are summarized in Table 1, including our case [5–10]. The median age of the patients was 63 (range 49–76); 86% were male patients. Amyloid A amyloidosis was reported in three patients, and amyloid light chain amyloidosis in the other three. The most frequent preoperative diagnosis was cholecystitis. However, additional patients were diagnosed with gallbladder tumors. Of the seven patients including our case, five did not exhibit gallbladder stones formation. Preoperative imaging findings showed variability and lacked specificity, as in our case. Some patients had underlying chronic renal failure, multiple myeloma, or a monoclonal gammopathy of undetermined significance. One patient who had a fatal outcome expired of renal failure and cirrhosis due to progressive systemic amyloidosis after surgery.

**Table 1:** Reported cases of gallbladder amyloidosis

Case number	author	Year	Age/sex	Preoperative diagnosis	Gallbladder stone	operation	Amyloid type	Underlying disease	Outcome
1	Kim	2003	63/M	Cholecystitis	−	OC	AA	Pulmonary TB	NS
2	Kwon	2007	63/F	Gallbladder Cancer	−	LC	AL	None	Alive
3	Tirotta	2011	74/M	Cholecystitis	−	OC	NS	None	NS
4	Um	2016	69/M	Cholecystitis	−	LC	AL	MGUS	Dead
5	Matsuda	2019	76/M	Cholecystitis	+	OC	AA	Bronchiectasis	NS
6	Hashmi	2021	49/M	Cholecystitis	+	Cholecystectomy	AA	CKD	NS
7	Our Case	2023	59/M	Gallbladder dyskinesia	−	LC	AL	MM	Alive

*NS* not stated, *OC* open cholecystectomy, *AA* amyloid A, *AL* amyloid light chain, *TB* tuberculosis, *CKD* chronic kidney disease, *MM* multiple myeloma, *MGUS* monoclonal gammopathy of undetermined significance

The patient underwent surgery for colic attacks due to gallbladder dyskinesia before developing cholecystitis. The patient had a history of multiple myeloma and cardiac amyloidosis. Abdominal ultrasonography revealed bile sludge without gallstones formation. After PTGBA, the patient continued to have a gallbladder effusion and recurrent right upper quadrant pain, leading to the suspicion of gallbladder contraction failure. Flopropione was markedly effective in relieving this symptom, leading to the diagnosis of gallbladder dyskinesia. Flopropione had anticholinergic properties and was thought to be significantly effective in the tension type of dyskinesia of the gallbladder [11]. Systemic amyloidosis is characterized by amyloid deposits in the heart, kidneys, and gastrointestinal tract [12]. As Table 1 presents, all patients with gallbladder amyloidosis have systemic amyloidosis. Therefore, the possibility of gallbladder amyloidosis was suspected preoperatively in this case.

Emergency cholecystectomy is recommended for acute cholecystitis when surgery is possible. According to the guidelines for acute cholecystitis, the mortality rate is generally less than 1% [13]. However, acute cholecystitis due to gallbladder amyloidosis carries the risk of coexisting organ damage other than that of the gallbladder and requires caution. Patients with cardiac amyloidosis are at risk of having difficult airways to secure due to macroglossia, hemodynamic instability due to diastolic dysfunction, decreased cardiac outputs, and serious arrhythmias due to conduction system dysfunctions with amyloid deposition in the myocardial fibers [14]. In addition, a significantly higher mortality rate has been reported in patients with heart failure who develop acute cholecystitis compared with others [15, 16]. Surgical complications that should be noted in patients with amyloidosis include hemorrhage. This is because amyloidosis is associated with many defects in platelet function and coagulation, including abnormal platelet aggregation, increased vascular fragility, factor IX and factor X deficiency, decreased alpha-2-plasmin inhibitor levels, and increased plasminogen levels [17, 18]. A high rate of liver biopsy-related bleeding requiring blood transfusion has been reported in patients with hepatic amyloidosis [19].

If gallbladder amyloidosis was not suspected, cholecystectomy may have been performed at the time of inflammatory exacerbation, which might lead to worsening of heart failure and increased bleeding. A preoperative diagnosis was also advantageous in that the patient could receive adequate support from other departments and the operation was safely performed while the patient's general condition was relatively stable.

In this case, the patient underwent a laparoscopic cholecystectomy on a standby basis. The patient's heart failure was stabilized by the intervention by a cardiologist, and the multiple myeloma was stabilized using chemotherapy. The patient underwent surgery after consultation with the Department of Cardiology and the Department of Clinical Oncology. Surgery could be performed before cholecystitis onset and was relatively safe with minimal blood loss without postoperative worsening of heart failure. The patient was discharged on postoperative day 5.

Two mechanisms are thought to be involved in the pathogenesis of gallbladder amyloidosis causing cholecystitis: (1) rapid edematous changes due to deposition of amyloid protein in the walls of microvessels and (2) vascular obstruction caused by deposited amyloid proteins, which results in contractile dysfunction of the gallbladder and bile retention [6, 20]. In this case, there was mild chronic inflammation and fibrosis in the gallbladder wall. Amyloid deposition was observed from the mucosal intrinsic layer to the submucosal layer but did not lead to ischemia. A new finding in this case was the presence of amyloid deposits in the peripheral nerves. To the best of our knowledge, this is the first case report of gallbladder amyloidosis in which amyloids were deposited in the peripheral nerves of the gallbladder. It is possible that amyloid deposition in the peripheral nerves and contractile dysfunction of the gallbladder have caused the dyskinesia symptoms in this case.

## Conclusion

In our case, gallbladder dyskinesia symptoms led us to suspect gallbladder amyloidosis, and we safely surgically treated the patient before cholecystitis onset. Since patients with amyloidosis are at a high surgical risk, the disease status of amyloidosis and indications and timing of surgery must be carefully considered.

## Data Availability

Not applicable.

## Abbreviations

ENGBD: Endoscopic naso-gallbladder drainage
ENBD: Endoscopic nasobiliary drainage
PTGBA: Percutaneous transhepatic gallbladder aspiration

## References

[1] Dember LM (2006). Amyloidosis-associated kidney disease. J Am Soc Nephrol.

[2] Merlini G, Bellotti V (2003). Molecular mechanisms of amyloidosis. N Engl J Med.

[3] Kyle RA, Gertz MA (1995). Primary systemic amyloidosis: clinical and laboratory features in 474 cases. Semin Hematol.

[4] Ichimata S, Hata Y, Nishida N (2021). Effects of sporadic transthyretin amyloidosis frequently on the gallbladder and the correlation between amyloid deposition in the gallbladder and heart: a forensic autopsy-based histopathological evaluation. Pathol Int.

[5] Tirotta D, Durante V (2011). Uncommon localization of amyloidosis in gallbladder: description of a case and brief literature review. Ann Hepatol.

[6] Matsuda S, Nishikata M, Takai K, Motoyoshi T, Yamashita Y, Kirishima T (2019). An unusual case of acute cholecystitis with amyloidosis: a case report and literature review. Intern Med.

[7] Kwon AH, Tsuji K, Yamada H, Okazaki K, Sakaida N (2007). Amyloidosis of the gallbladder mimicking gallbladder cancer. J Gastroenterol.

[8] Kim SH, Han JK, Lee KH, Won HJ, Kim KW, Kim JS (2003). Abdominal amyloidosis: spectrum of radiological findings. Clin Radiol.

[9] Um YJ, Kim HA, Jung JH, Cho H, Kang JK (2016). A case of amyloidosis presenting as chronic cholecystitis, misdiagnosed as polymyalgia rheumatica. Korean J Gastroenterol.

[10] Hashmi S, Munis A, Hoff RT, Kavin H, Ehrenpreis ED (2021). Secondary amyloidosis presenting as ischemic proctitis. Case Rep Gastrointest Med.

[11] Sasaki T, Kato D, Shinya S, Nakashima R, Shiwaku H, Yamashita K (2014). Successful treatment of gallbladder dyskinesia by laparascopic cholecystectomy: report of a case. Asian J Endosc Surg.

[12] Muchtar E, Dispenzieri A, Magen H, Grogan M, Mauermann M, McPhail ED (2021). Systemic amyloidosis from A (AA) to T (ATTR): a review. J Intern Med.

[13] Yokoe M, Hata J, Takada T, Strasberg SM, Asbun HJ, Wakabayashi G (2018). Tokyo Guidelines 2018: diagnostic criteria and severity grading of acute cholecystitis (with videos). J Hepatobiliary Pancreat Sci.

[14] Smith MA, Feinglass NG (2024). Perioperative implications of amyloidosis and amyloid cardiomyopathy: a review for anesthesiologists. J Clin Anesth.

[15] Marco-Martinez J, Elola-Somoza FJ, Fernandez-Perez C, Bernal-Sobrino JL, Azana-Gomez FJ, Garcia-Klepizg JL (2021). Heart failure is a poor prognosis risk factor in patients undergoing cholecystectomy: results from a Spanish data-based analysis. J Clin Med.

[16] Hall CM, Jupiter DC, Regner JL (2016). Newly diagnosed and decompensated congestive heart failure is associated with increased rates of pneumonia, reintubation, and death following laparoscopic cholecystectomy: a NSQIP database review of 143,761 patients. Int J Surg.

[17] Thompson CA, Kyle R, Gertz M, Heit J, Pruthi R, Pardanani A (2010). Systemic AL amyloidosis with acquired factor X deficiency: a study of perioperative bleeding risk and treatment outcomes in 60 patients. Am J Hematol.

[18] Mizutani AR, Ward CF (1990). Amyloidosis associated bleeding diatheses in the surgical patient. Can J Anaesth.

[19] Park MA, Mueller PS, Kyle RA, Larson DR, Plevak MF, Gertz MA (2003). Primary (AL) hepatic amyloidosis: clinical features and natural history in 98 patients. Medicine.

[20] Laurila JJ, Ala-Kokko TI, Laurila PA, Saarnio J, Koivukangas V, Syrjala H (2005). Histopathology of acute acalculous cholecystitis in critically ill patients. Histopathology.

